# Parallel evolution of site‐specific changes in divergent caribou lineages

**DOI:** 10.1002/ece3.4154

**Published:** 2018-05-15

**Authors:** Rebekah L. Horn, Adam J. D. Marques, Micheline Manseau, Brian Golding, Cornelya F. C. Klütsch, Ken Abraham, Paul J. Wilson

**Affiliations:** ^1^ Trent University Peterborough ON Canada; ^2^ Instituto Gulbenkian de Ciência Oeiras Portugal; ^3^ Science and Technology Environment and Climate Change Canada Ottawa ON Canada; ^4^ Natural Resources Institute University of Manitoba Winnipeg MB Canada; ^5^ Department of Biology McMaster University Hamilton ON Canada

**Keywords:** cytochrome‐*b*, functional diversity, nonsynonymous substitutions, *Rangifer*, TreeSAAP

## Abstract

The parallel evolution of phenotypes or traits within or between species provides important insight into the basic mechanisms of evolution. Genetic and genomic advances have allowed investigations into the genetic underpinnings of parallel evolution and the independent evolution of similar traits in sympatric species. Parallel evolution may best be exemplified among species where multiple genetic lineages, descended from a common ancestor, colonized analogous environmental niches, and converged on a genotypic or phenotypic trait. Modern North American caribou (*Rangifer tarandus*) originated from three ancestral sources separated during the Last Glacial Maximum (LGM): the Beringian–Eurasian lineage (BEL), the North American lineage (NAL), and the High Arctic lineage (HAL). Historical introgression between the NAL and the BEL has been found throughout Ontario and eastern Manitoba. In this study, we first characterized the functional differentiation in the cytochrome‐b (cytB) gene by identifying nonsynonymous changes. Second, the caribou lineages were used as a direct means to assess site‐specific parallel changes among lineages. There was greater functional diversity within the NAL despite the BEL having greater neutral diversity. The patterns of amino acid substitutions occurring within different lineages supported the parallel evolution of cytB amino acid substitutions suggesting different selective pressures among lineages. This study highlights the independent evolution of identical amino acid substitutions within a wide‐ranging mammal species that have diversified from different ancestral haplogroups and where ecological niches can invoke parallel evolution.

## 1 INTRODUCTION

The common occurrence of similar phenotypic or genotypic traits among sympatric species in similar environmental conditions is coined parallel evolution. The driving force behind parallel evolution is natural selection, as the probability of parallel mutational changes by random acts such as genetic drift is highly unlikely (Thompson, Taylor, & McPhail, 1997). Natural selection is often constrained by several factors, including evolutionary history and developmental restrictions (Elmer & Meyer, 2007); however, differences within and among species can arise from mutations on the same gene or different genes (Arendt & Reznick, 2008). The chance of parallel evolution occurring in natural populations depends on how many beneficial mutations are available from the ancestral sequence and the standing genetic variation present (Orr, 2005). It has been estimated using the expected probability of beneficial mutations that under natural selection, parallel evolution occurs two‐thirds of the time compared to a scenario of neutral evolution (Orr, 2005). The probability of parallel evolution arising from standing genetic variation increases when natural selection is strong and particularly for genes that have a large phenotypic effect and large population sizes. In this situation, parallel evolution can occur across multiple loci. If selection is weak and population sizes are small, then parallel evolution is expected at only a few loci (MacPherson & Nuismer, 2017).

Phenotypic or life history traits have been documented to evolve in parallel from several species, driven by similarities across geographically distinct habitats and similar selection pressures (Colosimo et al., 2005; Elmer, Kusche, Lehtonen, & Meyer, 2010; Johnson, 2001; Nosil, Crespi, & Sandoval, 2002; Palkovacs, Dion, Post, & Caccone, 2008; Reid, Herbelin, Bumbaugh, Selander, & Whittam, 2000; Thompson et al., 1997; Waples, Teel, Myers, & Marshall, 2004). Parallel evolution has also been detected at the level of ecotypes or morphs within species, with certain traits repeatedly arising in parallel due to similar environments or ecological niches (Foster, McKinnon, Steane, Potts, & Vaillancourt, 2007; Østbye et al., 2006; Perreault‐Payette et al., 2017; Taylor, Foote, & Wood, 1996). The recent advances in genomics have allowed for a deeper understanding of parallel evolution at the genomic level and to identify the specific genes responsible for the independent evolution and convergence of similar traits (Balanovsky et al., 2011; Wang et al., 2013; Wicker et al., 2009).

The identification of genes under selection can facilitate our understanding of parallel evolution, but functional diversity can often be hard to uncover in natural populations with limited genomic resources. Genes commonly under selection in the mitochondrial genome, such as cytochrome‐b (cytB) or cytochrome oxidase I (COI), are limited in studies of nonmodel organisms because they often do not provide the resolution for intraspecific diversity or for delineating population boundaries (Kvie, Heggenes, & Røed, 2016). Although conserved, these genes can undergo bouts of mutational change within species (Castoe, Jiang, Gu, Wang, & Pollock, 2008) leading to increased genetic diversity upon which evolutionary forces such as selection can act, particularly in heterogenous or extreme environments (Da Fonseca, Johnson, O'Brien, Ramos, & Antunes, 2008). This may lead to independent evolution of identical mutations that are retained in gene pools across the landscape where similar environmental conditions and selection pressures are present for the populations, thereby leading to regional parallel evolution. For example, parallel evolution of three populations of a dwarf ecotype that were each more closely related to the local tall ecotypes has been shown in the forest tree, *Eucalyptus globulus* (Foster et al., 2007). Alternatively, retention of different mutations in diverse environments indicates different selection pressures that ultimately lead to divergent evolution. This has been shown within species suggesting that the presence of functional differences at the genetic level can cause phenotypic and/or behavioral differences (Foote et al., 2011). Furthermore, introgressive hybridization, as an increasingly recognized evolutionary process for adding novel genomic components (Abbott et al., 2013; Hedrick, 2013; Seehausen, 2004), is another source where parallel changes would be predicted to occur on the introgressed genes; however, few studies have assessed signatures of parallel changes at such regions (Dowling et al., 2016; Meier et al., 2017).

North American caribou (*Rangifer tarandus*) demonstrate significant intraspecific variation with several subspecies, and ecotypes largely conforming to 12 recognized Designatable Units (DUs) having been identified based on ecological niches and calving strategies (COSEWIC 2011). One major identifier among ecotypes is displaying either sedentary or migratory behavior (COSEWIC 2011); for example, barren ground caribou undertake long annual migrations and can have seasonal home ranges as large as 111,000 km^2^ (COSEWIC 2011; McDevitt et al., 2009; Nagy, Wright, Slack, & Veitch, 2005; Yannic et al., 2014), whereas woodland caribou have a restricted home range size, with annual population home range sizes from 200 to 1200 km^2^ (Rettie & Messier, 2001) and in the summer can be as low as 17 km^2^ (Wittmer, McLellan, Serrouya, & Apps, 2007). Additional subspecies of *Rangifer* also occur throughout Eurasia, where they are named reindeer. The glacial cycles during the Quaternary and suitable refugia led to the evolution of three distinct genetic lineages of *Rangifer* across their Holarctic distribution: the Beringian Eurasian lineage (BEL), the North American lineage (NAL) (Cronin, Macneil, & Patton, 2005; Flagstad & Røed, 2003; Klütsch, Manseau, & Wilson, 2012; Røed, Ferguson, Crête, & Bergerud, 1991; Yannic et al., 2014), and a more recently identified High Arctic lineage (HAL) (Klütsch, Manseau, Anderson, Sinkins, & Wilson, 2017). The NAL, with proposed refugial origins south of the Laurentide ice sheet, is further genetically subdivided into three haplogroups, the likely result of multiple, distinct glacial refugia within North America during the Last Glacial Maximum (Klütsch et al., 2012). Genetic introgression of the NAL and the BEL likely occurred after glacial retreat in North America, about 11,500 years ago, as there are mixed BEL and NAL haplotypes present in many boreal caribou herds (Klütsch, Manseau, Trim, Polfus, & Wilson, 2016; Klütsch et al., 2012).

There have been several studies characterizing the genetic diversity and population differentiation of North American caribou at neutral loci (Flagstad & Røed, 2003; Klütsch et al., 2012, 2016; Kuhn, McFarlane, Groves, Mooers, & Shapiro, 2010; McDevitt et al., 2009; Weckworth, Musiani, McDevitt, Hebblewhite, & Mariani, 2012), but little work has been carried out to resolve differences among lineages at the level of functional genes that may convey adaptation to ecological niches or their behavior. Different selective pressures may exist across ecotypes and lineages, particularly in relation to energy requirements and demands facilitated by the mitochondrial genome for migratory or sedentary behavior. Our first objective was to characterize the functional genetic diversity of the cytB gene in divergent caribou and reindeer lineages. Previous genetic analyses of *Rangifer* using the cytB gene have shown greater genetic diversity in the BEL compared to the NAL (Cronin et al., 2005; Yannic et al., 2014); therefore, we predict greater functional diversity in the BEL. To test this hypothesis, we characterize nonsynonymous changes within cytB and test for significantly radical amino acid substitutions. Our second objective was to use the presence of two phylogenetic lineages (BEL and NAL), three sublineages (NAL1, NAL2, NAL3), and introgression between the BEL and the NAL to provide a means of testing for the parallel evolution of site‐specific changes within lineages that may inform on the various selective pressures unique to each lineage. We define parallel evolution as the occurrence of site‐specific changes that occur within ecotypes that may belong to different lineages or sublineages and those changes arose before lineages contacted after the glacial retreat. To test for parallel evolution, we reconstructed the phylogeny based on cytB to investigate whether mutations have evolved independently multiple times in caribou and reindeer lineages and when in time those mutations occurred.

## 2 MATERIALS AND METHODS

Cytochrome‐b haplotypes for *Rangifer tarandus* were retrieved from the National Center for Biotechnology Information (NCBI) from three independent studies (Cronin, Macneil, & Patton, 2006; Cronin et al., 2005; Yannic et al., 2014) representing samples from Eurasia and North America (Table S1). Additional samples from North America, originally reported in Klütsch et al. (2012), were sequenced at the cytB gene (Table S2). Using the Klütsch et al. (2012) samples, we can identify which mitochondrial control region haplogroup each sample belongs to, either the BEL or one of the three NAL haplogroups: NAL1, NAL2, or NAL3, originally defined by Klütsch et al. (2012). DNA was extracted by swabbing the mucosal layer of fecal pellets and the use of a Qiagen DNAeasy (Toronto, Ontario) extraction kit; for more detailed extraction methods, see Ball et al. (2007). A cytB‐specific primer pair (LGL766 5′‐GTT TAA TTA GAA TYT YAG CTT TGG G‐3′; LGL 765 5′‐GAA AAA CCA YCG TTG TWA TTC AAC T‐3′; Cronin, Shideler, Hechtel, Strobeck, and Paetkau (1999)) was used to amplify a 1,235 bp fragment of cytB. A set of internal sequencing primers (CytBRang‐IntF 5′‐GAA CTC CTC ATT CCA CCT‐3′; CytBRang‐IntR 5′‐TTA GTA CCT GCT CGG GAA C‐3′) was designed to verify substitutions occurring in the first 25 amino acids. Each PCR reaction consisted of 10X PCR buffer, 1.5 mmol/L MgCl_2_, 0.2 mmol/L DNTPs, 0.1 mg/ml BSA, 0.2 μmol/L of each primer, and 0.04 U/μl of *Taq*. Reactions were run in a thermocycler with the following conditions: 94°C for 5 min, 30 cycles of 94°C for 30 s, 56°C for 1 min, and 72°C for 30 s, and a final step of 72°C for 15 min. Amplified products were run on a QiAxcel Electrophoresis system for quantification, and all samples were diluted to 2.5 ng/100 base pairs. Amplified DNA was purified using the New England BioLabs Exonuclease I and Antarctic Phosphotase. The ABI BigDye Terminator sequencing reaction kit was used following the manufactures protocols and products were run on an ABI3730.

Sequence results were analyzed and aligned to the *Rangifer tarandus* mitochondrial genome reference sequence (NC_007703.1; Wada, Nakamura, Nishibori, & Yokohama, 2010) in Geneious v6.0.5 (Kearse et al., 2012). Ambiguous reads resulting from either low amplitude or double peaks were either resequenced or excluded from subsequent analyses. Unique haplotypes were identified using DnaSP v5 (Librado & Rozas, 2009) and all haplotypes were scanned manually for the presence of nonsynonymous substitutions. The ratio of nonsynonymous to synonymous substitutions (Ka/Ks) within the NAL and BEL caribou lineages was calculated with DnaSP v5 (Librado & Rozas, 2009). Tests for positive selection were performed with a gene‐wide and an individual‐site model using the BUSTED (Branch‐site Unrestricted Statistical Test for Episodic Diversification; Murrell et al., 2015) and MEME (Mixed Effects Model of Evolution; Murrell et al., 2012) methods, respectively, in the Datamonkey web‐suite of phylogenetic tools (Delport, Poon, Frost, & Pond, 2010; Pond & Frost, 2005; Pond, Frost, & Muse, 2005).

The total haplotype alignment, including all reindeer and caribou sequences retrieved from NCBI, and the newly generated caribou cytB sequences were tested for deviations from neutrality using Tajima's *D* statistic (Tajima, 1989). The accumulation of deleterious mutations in cytB can have severe repercussions for fitness (Hill, Chen, & Xu, 2014; Peischl, Dupanloup, Kirkpatrick, & Excoffier, 2013) and therefore, significant purifying selection was the expected outcome. Functional variants among the haplotypes were identified using TreeSAAP v3.2 (Woolley, Johnson, Smith, Crandall, & McClellan, 2003) which estimate the substitutions impact on the physiological properties of resulting proteins. The method defines physiological properties and categorizes change in protein physiology (*R*) in order of conserved to increasingly radical changes in physiology (McClellan et al., 2005). A preliminary analysis with all physiological properties and eight categories of radicality was used to identify putatively significant properties. Subsequent analysis used a sliding window to scan for significant radical substitutions (*R *≥ median *R*;* p *>* *0.95 with Bonferonni correction). Functional variants were considered putatively beneficial only when evidence suggested that the associated haplotype had persisted through time (McClellan & Ellison, 2010).

The evolutionary relationship among cytB haplotypes was assessed with a minimum evolution tree in MEGA v6.06 (Tamura, Stecher, Peterson, Filipski, & Kumar, 2013) using 1,000 bootstrap replicates. The substitution model was set to the Tamura‐Nei model (Tamura & Nei, 1993), as predicted by a corrected Akaike Information Criterion (AICc) analysis in jModel Test v6.02 (Darriba, Taboada, Doallo, & Posada, 2012; Guindon & Gascuel, 2003). As some nonsynonymous substitutions occur across genetic lineages or on different clades of the minimum evolution tree, a Bayesian phylogenetic analysis was used to give approximate dates for those nonsynonymous substitutions. In this way, it can be determined whether the substitutions occurred after the retreat of the ice sheet and in which genetic lineage the substitution first occurred. This analysis was performed in the program BEASTv1.8.4 (Drummond, Suchard, Xie, & Rambaut, 2012) using a strict clock model, the same substitution model as was estimated for the minimum evolution tree, and the caribou cytB‐specific mutation rate from Yannic et al. (2014). The Markov Monte Carlo Chain was run for 300,000,000 steps with sampling every 1,000 steps. The program Tracer v1.5 (Rambaut, Suchard, Xie, & Drummond, 2014) was used to assess convergence of the parameters and the program FigTree v1.4.3 (Rambaut, 2016) was used to visualize the resulting tree. A mtCR minimum joining haplotype network (Bandelt, Forster, & Röhl, 1999) was assembled using the mtCR haplotype data from Klütsch et al. (2012) in the program PopART (Leigh & Bryant, 2015). The nonsynonymous cytB substitutions were overlaid onto the mtCR network to visually assess evolutionary relationships among those sequences with nonsynonymous substitutions.

## 3 RESULTS

A total of 232 samples that have a known mtCR haplotype (Klütsch et al., 2012) were sequenced at the cytB gene. Of the 232 samples, 27 samples either failed sequencing or contained stretches of poor DNA quality and were not included in subsequent analyses or in the calculation of distinct haplotypes. Poor‐quality DNA sequences were scanned for amino acid substitutions at known sites; if a substitution was present and the sequence quality was good at the substitution site, the sample's location was recorded and used in the overall distribution of amino acid substitutions. There were 149 *Rangifer* cytB sequences (*n* = 59, Cronin et al., 2005; *n* = 14, Cronin et al., 2006; *n* = 76, Yannic et al., 2014) retrieved from NCBI (Table S1) and aligned with the cytB sequences from this study. After alignment, all cytB sequences were trimmed to 1,062 bp, a length that included the majority of the cytB gene except for the first 25 amino acids. A total of 144 unique cytB haplotypes were present, of which 27 were distinct from previously published cytB sequences (Table S2).

The Ka/Ks ratio for the BEL averaged 0.02 and ranged from 0.00 to 0.34 and the Ka/Ks ratio for the NAL averaged 0.23 and ranged from 0.00 to 0.97. Note that the HAL, which includes Peary caribou, had the same cytB haplotype as other barren‐ground samples (BEL); therefore, the HAL results will not be reported separately, and samples of this lineage will be included with the BEL. There were a total of 27 nonsynonymous substitutions; 12 were observed in only one individual and hence were assumed to be deleterious and likely to be removed quickly from a population (Kimura, 1983; Eyre‐Walker and Keightley 2007) and were therefore not included in further analyses. Of the nonsynonymous substitutions that were observed more than once, there were several that occurred in more than five individuals, including an Ile to Val substitution at amino acid position 19 (Ile19Val), a Met to Leu substitution at amino acid position 82 (Met82Leu), an Ala to Thr substitution at amino acid position 190 (Ala190Thr), a Leu to Met substitution at amino acid position 235 (Leu235Met), a Leu to Phe substitution at amino acid position 237 (Leu237Phe), a Leu to Met substitution at amino acid position 241 (Leu241Met), an Ala to Thr substitution at position 246 (Ala246Thr), and a Thr to Met substitution at position 309 (Thr309Met) (Table 1). Seven of the 16 nonsynonymous changes that occurred in more than one individual changed the polarity of the amino acid (Table 1). TreeSAAP was then used to identify those substitutions that had a significant radical change in the amino acids property. The Yannic et al. (2014) sequences were used as training data. An initial analysis was performed with no sliding window, eight categories of radicality, and all physiological properties. This initial run indicated that among the physiological properties, polarity, coil tendencies, and alpha‐helical tendencies were putatively significant. Subsequent reanalysis included these three aforementioned physiological properties as well as a sliding window of ten amino acids and five categories of radicality; these parameters were adjusted to reduce the probability of false positives (McClellan & Ellison, 2010). Two substitutions were predicted to incur significant (α = 0.05 with Bonferonni correction) physiological changes in alpha‐helical tendencies, Ile19Val (*R *=* *4; *p *=* *9.3E‐5) and Ala246Thr (*R *=* *4; *p *=* *9.4E‐5) (Table 2) and were found in the NAL1, NAL2 and the BEL‐introgressed lineages. For the full cytB alignment, Tajima's *D* indicated that cytB deviated from strict neutrality among *Rangifer tarandus* (*D *=* *−1.78; *p *<.01). Tajima's *D* was also calculated for the NAL and BEL lineages separately. The NAL had a near significant negative value for *D* (*D *=* *−1.40; *p *>.05), while the BEL had a significantly negative *D* value (*D *=* *−2.15; *p *<.001). Tests for gene‐wide and site‐specific positive selection did not indicate significant evidence of positive selection across or within the cytB gene.

**Table 1:** The amino acid site of each nonsynonymous substitution, whether the substitution results in a change in polarity and the presence or absence of substitutions in each haplogroups, where BEL‐IN represents the introgressed BEL

Site	Substitution	Polarity	BEL	BEL‐IN	NAL1	NAL2	NAL3
19	**Ile‐Val**	Yes		X			
56	Thr‐Ser	No	X				
82	Met‐Leu	No					X
187	Phe‐Leu	No	X				
188	Ile‐Met	No	X				
190	Ala‐Thr	Yes	X		X		
212	Pro‐Ser	Yes	X				X
235	Leu‐Met	No	X				
237	Leu‐Phe	No	X				X
241	Leu‐Met	No					X
246	**Ala‐Thr**	Yes	X		X	X	
257	Thr‐Ile	Yes	X				X
295	Val‐Ile	No	X				
296	Ser‐Leu	Yes	X				
309	Thr‐Met	Yes	X		X		X
371	Thr‐Met	Yes	X				

The substitutions in bold are values identified as having a significant radical change in the protein function.

**Table 2:** The number of cytochrome‐b (cytB) haplotypes, control region (mtCR) haplotypes, unique proteins present (Proteins), and the proportion of individuals that have the Ile19Val or Ala246Thr substitution in each genetic lineage (BEL‐IN, introgressed BEL) for newly sequenced samples

Lineage	Sample Size	cytB Hap	mtCR Hap	Proteins	Ile19Val	Ala246Thr
NAL1	26	4	7	3	–	0.85
NAL2	70	5	13	1	–	0.30
NAL3	52	9	8	6	–	–
NAL	148	17	28	8	–	0.29
BEL	48	22	27	3	–	–
BEL‐IN	9	4	4	1	0.44	–

**Figure 1:**
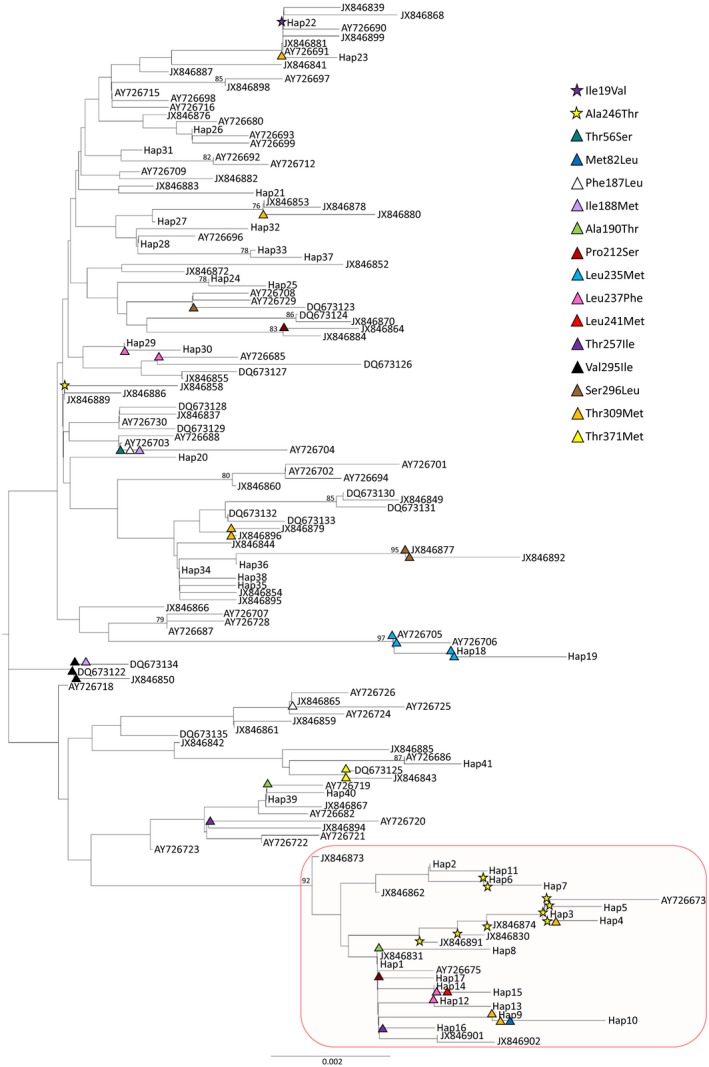
A minimum evolution tree of all cytB haplotypes with bootstrap support greater than 75% on the node labels. The red box highlights those haplotypes from the North American Lineage (NAL) ancestry. The nonsynonymous changes are overlaid onto corresponding branches with those changes identified as having a significant radical effect on the protein structure represented by a star

The minimum evolution tree delineated the BEL from the NAL, concordant with cytB phylogenies reported for caribou (Cronin et al., 2005, 2006; Yannic et al., 2014) (Figure 1). The NAL cluster had far fewer haplotypes (*n* = 27) compared to the BEL cluster (*n* = 117). The nonsynonymous amino acid substitutions were mapped onto the branches of the minimum evolution tree. The NAL cluster had eight nonsynonymous changes, with two of those changes exclusive to the NAL (Met82Leu, Leu241Met) (Figure 1). The NAL cluster contained all the Ala246Thr substitution (identified as causing a significantly radical protein‐level change according to TreeSAAP), except for one GenBank individual of unknown origin. The Ala246Thr substitution is located on two different clades of the tree within the NAL and sequences ancestral to those did not have the substitution (Figure 1). There were three instances within the BEL cluster in which nonsynonymous changes were found exclusively associated with a clade of samples, including the Leu235Met, Val295Ile, and Thr371Met substitutions (Figure 1). The Leu235Met substitution was found in barren‐ground caribou samples from the Qamanirjuaq herd in Manitoba and Nunavut and the Bluenose herd in the northwest Territories. The cluster of samples containing the Val295Ile substitution is *Rangifer* samples from Alaska. Location data could be retrieved for only one of the three samples associated with the Thr371Met substitution and it was a reindeer sample from Scandinavia. One nonsynonymous substitution (Ile19Val) was found within introgressed BEL caribou (e.g., boreal caribou that have a BEL lineage), specifically from Woodland Caribou Provincial Park, Ontario.

**Figure 2:**
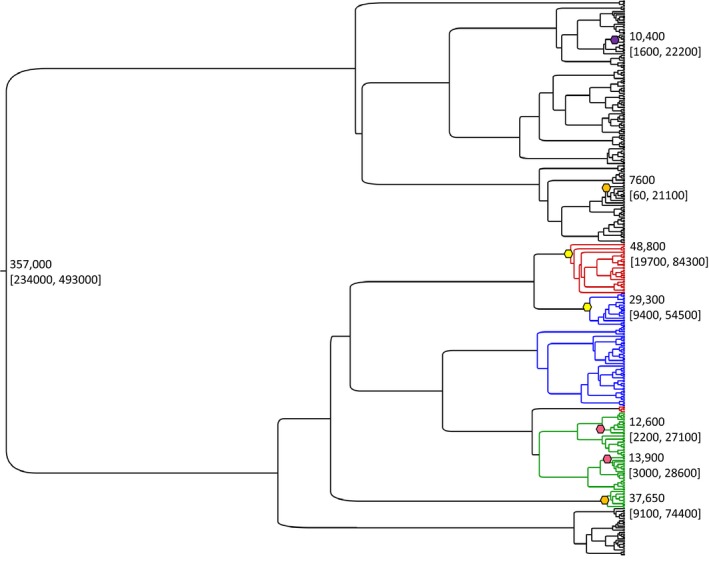
A Bayesian phylogenetic tree of all cytB sequences, including those retrieved from GenBank. The tree root age with 95% HPDI in brackets is displayed on the root node. The colored hexagon shapes are located on those nodes in the tree in which a nonsynonymous substitution occurred that could be dated (date in years before present with 95% HPDI to the right of each symbol). The purple symbol represents the Ile19Val substitution, orange is the Thr309Met substitution, yellow is the Ala246Thr substitution, and pink is the Leu237Phe substitution. The clades are color coded by the haplogroup: red, NAL1; blue, NAL2; green, NAL3; black,BEL

Based on the results of the minimum evolution tree and the frequency of occurrence, four nonsynonymous substitutions were dated using the Bayesian phylogenetic tree: Ile19Val, Leu237Phe, Ala246Thr, and Thr309Met. The Ile19Val substitution was found in boreal caribou with a BEL history; the occurrence of the substitution was dated to 10,400 years before present (95% HPDI: 1600, 22200), close to the end of the Last Glacial Maximum and the retreat of the Laurentian ice sheet in North America (Figure 2). The other three substitutions are found in both the NAL and BEL and Ala246Thr is found in both the NAL1 and NAL2. The Bayesian tree indicated that the Leu237Phe, Ala246Thr, and Thr309Met substitutions occurred in the NAL before their occurrence in the BEL (Figure 2). The occurrence of the Leu237Phe and Ala246Thr substitutions in the BEL lineage were estimated at zero, indicating the age of the node could not be estimated, whereas the Leu237Phe substitution was estimated to have occurred in two different NAL clades at 12,600 (95% HPDI: 2200, 27100) and 13,900 years before present (95% HPDI: 3000, 28600) (Figure 2). The occurrence of the Ala246Thr substitution was estimated to have occurred about 48,800 years before present (95% HPDI: 19700, 84300) in the NAL1 lineage and at 29,300 years before present (95% HPDI: 9400, 54500) in the NAL2 lineage, although confidence intervals overlap (Figure 2). This dates the Ala246Thr substitution as having arisen before the Last Glacial Maximum. The Thr309Met substitution was dated to approximately 37,650 years before present (95% HPDI: 9100, 74400) in the NAL and at 7,600 years before present (95% HPDI: 60, 21100) in the BEL (Figure 2).

**Figure 3:**
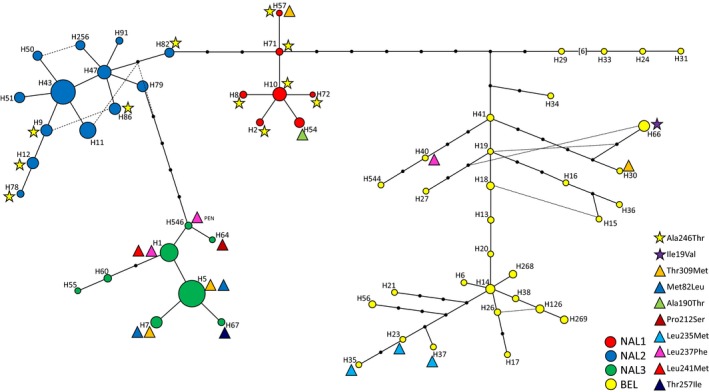
A minimum spanning network of the mitochondrial control region (mtCR) haplotypes that correspond to each sample sequenced in the present study at the cytB gene. The size of each circle is proportional to the number of sequences with that haplotype, the small black dots are hypothetical haplotypes, each line represents one mutational step, and the dashed lines are alternative connections between haplotypes. Haplotype number (H) corresponds to the mtCR haplotype designation by Klütsch et al. (2012) and circles are colored based on the haplogroup. CtyB nonsynonymous changes are labeled by a triangle next to each haplotype with those changes identified as having a significant radical effect on the protein structure represented by a star

The minimum joining network was constructed with the mtCR haplotypes, and cytB nonsynonymous changes were mapped onto the network (Figure 3). For this analysis, the GenBank samples were excluded as there was no accompanying mtCR haplotype for these samples. The mtCR network separated the BEL from the NAL by several mutational steps as well as delineated the three sublineages within the NAL: NAL1, NAL2, and NAL3 (Klütsch et al., 2012) (Figure 3). Nine of the nonsynonymous changes are present in the NAL cluster compared to four in the BEL. Within the NAL, there was one nonsynonymous change in NAL2 (Ala246Thr), three in NAL1 (Ala246Thr, Ala190Thr, Thr309Met), and six in NAL3 (Met82Leu, Pro212Ser, Leu237Phe, Leu241Met, Thr257Ile, Thr309Met) (Figure 3, Table 1). Three of the nonsynonymous changes are found within different lineages: Ala246Thr is found in NAL1 and NAL2, Leu237Phe is found in NAL3 and BEL, and Thr309Met is found in NAL1, NAL3, and BEL. A further examination of the Thr309Met substitution reveals that within the NAL3 cluster, it is only associated with individuals of the eastern‐migratory caribou ecotype sampled in northern Ontario and Manitoba and within the BEL cluster, with a haplotype (H30) that was from a barren‐ground caribou sampled in northern Manitoba. Of all the nonsynonymous changes, only two are exclusive to a mtCR haplotype: Ile19Val is associated with mtCR haplotype H66 and Leu241Met is associated with mtCR haplotype H1. The remaining nonsynonymous changes occur within cytB sequences associated with multiple mtCR haplotypes.

## 4 DISCUSSION

The first objective was to detect functional differences among caribou lineages in the cytB region of the mitochondria. Despite the BEL having a greater number of cytB haplotypes (Cronin et al., 2005, 2006; Yannic et al., 2014), the NAL had a proportionally large number of nonsynonymous amino acid substitutions and thereby functional diversity compared to the BEL (Table 2). The second objective was to use phylogenetic methods to test for the parallel evolution of nonsynonymous changes among genetic lineages. The Ala246Thr substitution arose independently in the NAL1, the NAL2, and the BEL suggesting parallel formation and retention of this substitution among haplogroups. Phylogenetic dating of this substitution suggests the occurrence in the NAL is older (i.e., before the LGM) compared to the occurrence in the BEL. One nonsynonymous change (Ile19Val) was only observed in introgressed BEL samples from Ontario and was dated to have occurred after the LGM, and while not an example of parallel change within this dataset, does represent a functional change specific to the introgressed mtDNA.

### 4.1 Evidence of parallelism

When small fractions of an ancestral population colonize new areas, the greater the background level of standing genetic variation, the higher likelihood for the divergence and fixation of novel genotypes or phenotypes that may only occur at low levels in the ancestral source (Elmer & Meyer, 2007). In caribou, the BEL likely represents the ancestral source for North American caribou and it is characterized by a greater level of genetic diversity compared to the NAL. Most of the nonsynonymous changes present in the cytB gene are found in the BEL; however, only in low frequencies. The same nonsynonymous changes are driven to a higher frequency in the NAL, which can be due to a variety of factors including neutral and selective processes (Nielsen, 2005). It was also estimated that the substitutions in the NAL are older indicating these mutational changes occurred before the NAL and BEL lineages could have become admixed (i.e., after the Last Glacial Maximum) and likely arose while the NAL was separated by the North American ice sheet and have been retained. For example, the Thr309Met substitution is found in eastern‐migratory animals located in northern Ontario and Manitoba and is estimated to have evolved in those lineages almost 38,000 years before present, before the LGM. A parallel change at the same amino acid position is noted in an individual from the Qamanirjuaq herd (barren‐ground caribou), which can overlap with the eastern‐migratory range during the winter months (COSEWIC 2011). The substitution in this individual is predicted to have occurred about 7,600 years before present, after the retreat of the North American ice sheet. Introgression between these lineages at that time (Klütsch et al., 2016) may have been the driver of the parallel evolution of this nonsynonymous change. Amino acid substitutions among many species classes overwhelmingly arise in parallel (Rokas & Carroll, 2008), and the parallel evolution of amino acid substitutions has been documented in a wide variety of species including mammals (Stewart, Schilling, & Wilson, 1987; Yeager & Hughes, 1999), fish (Jost et al., 2008; Yokoyama & Yokoyama, 1990), birds (Kornegay, Schilling, & Wilson, 1994), and amphibians (Malyarchuk et al., 2010).

The most widespread example of parallel evolution is the Ala246Thr substitution. The Ala246Thr substitution, based on the topology of the phylogenetic tree arose twice within the NAL, in two different haplogroups (NAL1 and NAL2) and one occurrence from a sample belonging to the BEL. It has been suggested that the NAL1 and NAL2 haplogroups were isolated in separate glacial refugia during the LGM, in the eastern and western United States, respectively (Klütsch et al., 2012). The presence of multiple glacial refugia for wide‐ranging and highly migratory mammal species has been shown previously (Shafer, Cullingham, Côté, & Coltman, 2010; Sim, Hall, Jex, Hegel, & Coltman, 2016), which supports the assertion of different glacial refugia for NAL1 and NAL2. There are two scenarios that could likely explain the widespread presence of the Ala246Thr substitution in the NAL1 and NAL2 haplogroups. First, the mutation may be present in the ancestral population in low frequency, but driven to fixation after diversification of a new lineage(s) in a novel environment (Elmer & Meyer, 2007). The Ala246Thr substitution is found in one individual from the BEL; the geographic origin of that sample is unknown, but this substitution in the BEL occurred too recently to be aged using the Bayesian phylogenetic method suggesting this is not the ancestral source of this substitution. If this were true, it would be expected that most, if not all the individuals belonging to the NAL1 and NAL2 lineages would possess this mutation due to the proliferation of the substitution from a small subset of the ancestral population. The second scenario is that this nonsynonymous change arose in parallel in the NAL1 and the NAL2 haplogroups. This scenario is more likely because first, the nonsynonymous changes are located on different branches of the tree with ancestral‐type sequences at the nodes of these branches. Second, during sequence evolution, amino acid changes occur at single amino acid residues, two‐amino acid, and three‐amino acid motifs (Yeager & Hughes, 1999). If the nonsynonymous change resulted from shared ancestry, there should be an equal number of one, two and three‐amino acid residue changes. Conversely, a larger number of single amino acid polymorphisms are indicative of parallel evolution, as these types of changes are more easily evolved (Yeager & Hughes, 1999). We observe one amino acid change within the cytB region, supporting the evidence for parallel evolution at the amino acid level within caribou.

### 4.2 Divergence within and among lineages

Almost half (44%) of the nonsynonymous changes observed in caribou changed the amino acid's polarity and two theoretically cause changes to the proteins physicochemical properties. The amino acid substitutions associated with changes in the protein's alpha‐helical tendencies (Branden & Tooze, 1999) were found associated with the boreal and eastern‐migratory caribou ecotypes. Cytochrome‐b functions as a proton pump formed by an alpha‐helical channel along the inner membrane of the mitochondria (Da Fonseca et al., 2008). It cannot be determined based on the available data if these amino acid changes correlate to changes in the function of the protein or if the change is neutral (Storz, 2016). Based on the broad distribution and lineage consistency of these significant changes, this could suggest that these substitutions represent true functional variants between caribou lineages; however, additional molecular biological based analysis would be required to confirm this as there was no statistical evidence for positive selection. Barren‐ground caribou (BEL) undertake large annual migrations (COSEWIC 2011; McDevitt et al., 2009; Yannic et al., 2014) which would reasonably entail high metabolic demands. Optimal efficiency may therefore be favorable among migrating subspecies which is supported by the significant purifying selection as indicated by Tajima's *D*. Radical functional variants in cytB may arise through a relaxing of selective pressures (Gering, Opazo, & Storz, 2009; McClellan et al. 2011) or when inefficiencies confer a benefit (Ballard & Pichaud, 2014). Woodland caribou are more sedentary compared to barren‐ground caribou (McDevitt et al., 2009), and different metabolic demands may allow selectively neutral or even beneficial functional variants to have evolved repeatedly. To infer causality between the substitutions and protein function among caribou ecotypes, additional work beyond the scope of this study is needed. Genome wide studies are beginning to reveal that there are several hundred genes that may be responsible for phenotypic differences among ecotypes (Bernatchez et al., 2010) and therefore, genome wide scans will likely be necessary to completely elucidate the adaptive genetic differences among caribou ecotypes.

## 5 CONCLUSIONS

Overall, we observed high levels of genetic diversity at the cytB gene in the BEL haplogroup of caribou, but greater functional diversity in the NAL compared to the BEL due to the parallel evolution and retention of lineage‐specific amino acid substitutions. Despite the poor resolution of the cytB gene for phylogenetic inferences of caribou (Kvie et al., 2016), variability in protein diversity is present and provides important information regarding the evolution of lineages and potential selection to the local environment. This may have important implications for the future protection and management of caribou, particularly when trying to determine genetically distinct units, as the difference in functional variability might need to be considered in conjunction with neutral genetic structure. This study demonstrates that despite belonging to the same evolutionary lineage, environment and/or location can drive the parallel evolution of nonsynonymous changes within functional genes, adding a new aspect to consider when assigning management criteria, such as designatable units.

## CONFLICT OF INTEREST

None declared.

## AUTHOR CONTRIBUTIONS

AM and CFCK were responsible for DNA sequencing. RLH and AM performed data analysis. MM, KA, and PJW were supervisors on the project. AM, RLH, MM, and PJW wrote the manuscript. CFCK, BG, and AM provided manuscript edits.

## References

[ece34154-bib-0001] Abbott, R. , Albach, D. , Ansell, S. , Arntzen, J. W. , Baird, S. J. , Bierne, N. , … Butlin, R. K. (2013). Hybridization and speciation. Journal of Evolutionary Biology, 26, 229–246. https://doi.org/10.1111/j.1420-9101.2012.02599.x 23323997 10.1111/j.1420-9101.2012.02599.x

[ece34154-bib-0002] Arendt, J. , & Reznick, D. (2008). Convergence and parallelism reconsidered: What have we learned about the genetics of adaptation? Trends in Ecology and Evolution, 23, 26–32. https://doi.org/10.1016/j.tree.2007.09.011 18022278 10.1016/j.tree.2007.09.011

[ece34154-bib-0003] Balanovsky, O. , Dibirova, K. , Dybo, A. , Mudrak, O. , Frolova, S. , Pocheshkhova, E. , … Kuznetsova, M. (2011). Parallel evolution of genes and languages in the Caucasus region. Molecular Biology and Evolution, 28, 2905–2920. https://doi.org/10.1093/molbev/msr126 21571925 10.1093/molbev/msr126 PMC3355373

[ece34154-bib-0004] Ball, M. C. , Pither, R. , Manseau, M. , Clark, J. , Petersen, S. D. , Kingston, S. , … Wilson, P. (2007). Characterization of *target* nuclear DNA from faeces reduces technical issues associated with the assumptions of low‐quality and quantity template. Conservation Genetics, 8, 577–586. https://doi.org/10.1007/s10592-006-9193-y

[ece34154-bib-0005] Ballard, J. W. O. , & Pichaud, N. (2014). Mitochondrial DNA: More than an evolutionary bystander. Functional Ecology, 28, 218–231. https://doi.org/10.1111/1365-2435.12177

[ece34154-bib-0006] Bandelt, H. J. , Forster, P. , & Röhl, A. (1999). Median‐joining networks for inferring intraspecific phylogenies. Molecular Biology and Evolution, 16, 37–48. https://doi.org/10.1093/oxfordjournals.molbev.a026036 10331250 10.1093/oxfordjournals.molbev.a026036

[ece34154-bib-0007] Bernatchez, L. , Renaut, S. , Whiteley, A. R. , Derome, N. , Jeukens, J. , Landry, L. , … St‐Cyr, J. (2010). On the origin of species: Insights from the ecological genomics of lake whitefish. *Philos* . Philosophical Transactions of the Royal Society B: Biological Sciences, 365, 1783–1800. https://doi.org/10.1098/rstb.2009.0274 10.1098/rstb.2009.0274 PMC2871888 20439281

[ece34154-bib-0008] Branden, C. L. , & Tooze, J. (1999). Introduction to protein structure, 2nd ed New York: Garland Publishing.

[ece34154-bib-0009] Castoe, T. A. , Jiang, Z. J. , Gu, W. , Wang, Z. O. , & Pollock, D. D. (2008). Adaptive evolution and functional redesign of core metabolic proteins in snakes. PLoS ONE, 3, e2201 https://doi.org/10.1371/journal.pone.0002201 18493604 10.1371/journal.pone.0002201 PMC2376058

[ece34154-bib-0010] Colosimo, P. F. , Hosemann, K. E. , Balabhadra, S. , Villarreal, G. Jr , Dickson, M. , Grimwood, J. , … Kingsley, D. M. (2005). Widespread parallel evolution in sticklebacks by repeated fixation of ectodysplasin alleles. Science, 307, 1928–1933. https://doi.org/10.1126/science.1107239 15790847 10.1126/science.1107239

[ece34154-bib-0011] COSEWIC (2011). Designatable units for caribou (Rangifer tarandus) in Canada. Ottawa, ON: Committee on the Status of Endangered Wildlife in Canada.

[ece34154-bib-0012] Cronin, M. A. , Macneil, M. D. , & Patton, J. C. (2005). Variation in mitochondrial DNA and microsatellite DNA in caribou (*Rangifer tarandus*) in North America. Journal of Mammalogy, 86, 495–505. https://doi.org/10.1644/1545-1542(2005)86[495:VIMDAM]2.0.CO;2

[ece34154-bib-0013] Cronin, M. A. , Macneil, M. D. , & Patton, J. C. (2006). Mitochondrial DNA and microsatellite DNA variation in domestic reindeer (*Rangifer tarandus tarandus*) and relationships with wild caribou (*Rangifer tarandus granti*,* Rangifer tarandus groenlandicus*, and *Rangifer tarandus caribou*). Journal of Heredity, 97, 525–530. https://doi.org/10.1093/jhered/esl012 16837563 10.1093/jhered/esl012

[ece34154-bib-0014] Cronin, M. , Shideler, R. , Hechtel, J. , Strobeck, C. , & Paetkau, D. (1999). Genetic relationships of grizzly bears (*Ursus arctos*) in the Prudhoe Bay region of Alaska: Inference from microsatellite DNA, mitochondrial DNA, and field observations. Journal of Heredity, 90, 622–628. https://doi.org/10.1093/jhered/90.6.622 10589512 10.1093/jhered/90.6.622

[ece34154-bib-0015] da Fonseca, R. R. , Johnson, W. E. , O'Brien, S. J. , Ramos, M. J. , & Antunes, A. (2008). The adaptive evolution of the mammalian mitochondrial genome. BMC Genomics, 9, 1–22. 18318906 10.1186/1471-2164-9-119 PMC2375446

[ece34154-bib-0016] Darriba, D. , Taboada, G. L. , Doallo, R. , & Posada, D. (2012). jModelTest 2: More models, new heuristics and parallel computing. Nature Methods, 9, 772 https://doi.org/10.1038/nmeth.2109 10.1038/nmeth.2109 PMC4594756 22847109

[ece34154-bib-0017] Delport, W. , Poon, A. F. , Frost, S. D. W. , & Pond, S. L. K. (2010). Datamonkey 2010: A suite of phylogenetic analysis tools for evolutionary biology. Bioinformatics, 26, 2455–2457. https://doi.org/10.1093/bioinformatics/btq429 20671151 10.1093/bioinformatics/btq429 PMC2944195

[ece34154-bib-0018] Dowling, T. E. , Markle, D. F. , Tranah, G. J. , Carson, E. W. , Wagman, D. W. , & May, B. P. (2016). Introgressive hybridization and the evolution of lake‐adapted Catostomid fishes. PLoS ONE, 11, e0149884 https://doi.org/10.1371/journal.pone.0149884 26959681 10.1371/journal.pone.0149884 PMC4784955

[ece34154-bib-0019] Drummond, A. J. , Suchard, M. A. , Xie, D. , & Rambaut, A. (2012). Bayesian phylogenetics with BEAUti and the BEAST 1.7. Molecular Biology and Evolution, 29, 1969–1973. https://doi.org/10.1093/molbev/mss075 22367748 10.1093/molbev/mss075 PMC3408070

[ece34154-bib-0020] Elmer, K. R. , Kusche, H. , Lehtonen, T. K. , & Meyer, A. (2010). Local variation and parallel evolution: Morphological and genetic diversity across a species complex of neotropical crater lake cichlid fishes. Philosophical Transactions of the Royal Society B: Biological Sciences, 365, 1763–1782. https://doi.org/10.1098/rstb.2009.0271 10.1098/rstb.2009.0271 PMC2871887 20439280

[ece34154-bib-0500] Elmer, K. R. , & Meyer, A. (2011). Adaptation in the age of ecological genomics: Insights from parallelism and convergence. Trends in Ecology & Evolution, 26, 298–306. https://doi.org/10.1016/j.tree.2011.02.008 21459472 10.1016/j.tree.2011.02.008

[ece34154-bib-0021] Eyre‐Walker, A. , & Keightley, P. D. (2007). The distribution of fitness effects of new mutations. Nature Review. Genetics, 8, 610–618. https://doi.org/10.1038/nrg2146 17637733 10.1038/nrg2146

[ece34154-bib-0022] Flagstad, Ø. , & Røed, K. H. (2003). Refugial origins of reindeer (*Rangifer tarandus* L.) inferred from mitochondrial DNA sequences. Evolution, 57, 658–670. https://doi.org/10.1111/j.0014-3820.2003.tb01557.x 12703955 10.1111/j.0014-3820.2003.tb01557.x

[ece34154-bib-0023] Foote, A. D. , Morin, P. A. , Durban, J. W. , Pitman, R. L. , Wade, P. , Willerslev, E. , … da Fonseca, R. R. (2011). Positive selection on the killer whale mitogenome. Biology Letters, 7, 116–118. https://doi.org/10.1098/rsbl.2010.0638 20810427 10.1098/rsbl.2010.0638 PMC3030902

[ece34154-bib-0024] Foster, S. A. , McKinnon, G. E. , Steane, D. A. , Potts, B. M. , & Vaillancourt, R. E. (2007). Parallel evolution of dwarf ecotypes in the forest tree *Eucalyptus globulus* . New Phytologist, 175, 370–380. https://doi.org/10.1111/j.1469-8137.2007.02077.x 17587385 10.1111/j.1469-8137.2007.02077.x

[ece34154-bib-0025] Gering, E. J. , Opazo, J. C. , & Storz, J. F. (2009). Molecular evolution of cytochrome b in high‐ and low‐altitude deer mice (genus Peromyscus). Heredity, 102, 226–235. https://doi.org/10.1038/hdy.2008.124 19107138 10.1038/hdy.2008.124 PMC4409697

[ece34154-bib-0026] Guindon, S. , & Gascuel, O. (2003). A simple, fast and accurate method to estimate large phylogenies by maximum‐likelihood. Systematic Biology, 52, 696–704. https://doi.org/10.1080/10635150390235520 14530136 10.1080/10635150390235520

[ece34154-bib-0027] Hedrick, P. W. (2013). Adaptive introgression in animals: Examples and comparison to new mutation and standing variation as sources of adaptive variation. Molecular Ecology, 22, 4606–4618. https://doi.org/10.1111/mec.12415 23906376 10.1111/mec.12415

[ece34154-bib-0028] Hill, J. H. , Chen, Z. , & Xu, H. (2014). Selective propagation of functional mitochondrial DNA during oogenesis restricts the transmission of a deleterious mitochondrial variant. Nature Genetics, 46, 389–392. https://doi.org/10.1038/ng.2920 24614072 10.1038/ng.2920 PMC3976679

[ece34154-bib-0029] Johnson, J. B. (2001). Adaptive life‐history evolution in the livebearing fish *Brachyrhaphis rhabdophora*: Genetic basis for parallel divergence in age and size at maturity and a test of predator‐induced plasticity. Evolution, 55, 1486–1491. https://doi.org/10.1111/j.0014-3820.2001.tb00668.x 11525470 10.1111/j.0014-3820.2001.tb00668.x

[ece34154-bib-0030] Jost, M. C. , Hillis, D. M. , Lu, Y. , Kyle, J. W. , Fozzard, H. A. , & Zakon, H. H. (2008). Toxin‐resistant sodium channels: Parallel adaptive evolution across a complete gene family. Molecular Biology and Evolution, 25, 1016–1024. https://doi.org/10.1093/molbev/msn025 18258611 10.1093/molbev/msn025 PMC2877999

[ece34154-bib-0031] Kearse, M. , Moir, R. , Wilson, A. , Stones‐Havas, S. , Cheung, M. , Sturrock, S. , … Thierer, T. (2012). Geneious Basic: An integrated and extendable desktop software platform for the organization and analysis of sequence data. Bioinformatics, 28, 1647–1649. https://doi.org/10.1093/bioinformatics/bts199 22543367 10.1093/bioinformatics/bts199 PMC3371832

[ece34154-bib-0032] Kimura, M. (1983). Rare Variant Alleles in the Light of the Neutral Theory. Molecular Biology and Evolution, 1, 84–93. 6599962 10.1093/oxfordjournals.molbev.a040305

[ece34154-bib-0033] Klütsch, C. F. C. , Manseau, M. , Anderson, M. , Sinkins, P. , & Wilson, P. J. (2017). Evolutionary reconstruction supports the presence of a Pleistocene polar refugium for a large mammal species. Journal of Biogeography, 44, 386–396.

[ece34154-bib-0034] Klütsch, C. F. C. , Manseau, M. , Trim, V. , Polfus, J. , & Wilson, P. J. (2016). The eastern migratory caribou: The role of genetic introgression in ecotype evolution. Royal Society Open Science, 3, 150469 https://doi.org/10.1098/rsos.150469 26998320 10.1098/rsos.150469 PMC4785971

[ece34154-bib-0035] Klütsch, C. F. C. , Manseau, M. , & Wilson, P. J. (2012). Phylogeographical analysis of mtDNA data indicates postglacial expansion from multiple glacial refugia in woodland caribou (*Rangifer tarandus caribou*). PLoS ONE, 7, 1–10. 10.1371/journal.pone.0052661 PMC3528724 23285137

[ece34154-bib-0036] Kornegay, J. R. , Schilling, J. W. , & Wilson, A. C. (1994). Molecular adaptation of a leaf‐eating bird: Stomach lysozyme of the hoatzin. Molecular Biology and Evolution, 11, 921–928. 7815930 10.1093/oxfordjournals.molbev.a040173

[ece34154-bib-0037] Kuhn, T. S. , McFarlane, K. A. , Groves, P. , Mooers, A. Ø. , & Shapiro, B. (2010). Modern and ancient DNA reveal recent partial replacement of caribou in the southwest Yukon. Molecular Ecology, 19, 1312–1323. https://doi.org/10.1111/j.1365-294X.2010.04565.x 20196813 10.1111/j.1365-294X.2010.04565.x

[ece34154-bib-0038] Kvie, K. S. , Heggenes, J. , & Røed, K. H. (2016). Merging and comparing three mitochondrial markers for phylogenetic studies of Eurasian reindeer (*Rangifer tarandus*). Ecology and Evolution, 6, 4347–4358. https://doi.org/10.1002/ece3.2199 27386080 10.1002/ece3.2199 PMC4893353

[ece34154-bib-0039] Leigh, J. W. , & Bryant, D. (2015). POPART: Full‐feature software for haplotype network construction. Methods in Ecology and Evolution, 6, 1110–1116. https://doi.org/10.1111/2041-210X.12410

[ece34154-bib-0040] Librado, P. , & Rozas, J. (2009). DnaSP v5: A software for comprehensive analysis of DNA polymorphism data. Bioinformatics, 25, 1451–1452. https://doi.org/10.1093/bioinformatics/btp187 19346325 10.1093/bioinformatics/btp187

[ece34154-bib-0041] MacPherson, A. , & Nuismer, S. L. (2017). The probability of parallel genetic evolution from standing genetic variation. Journal of Evolutionary Biology, 30, 326–337. https://doi.org/10.1111/jeb.13006 27801996 10.1111/jeb.13006

[ece34154-bib-0042] Malyarchuk, B. , Derenko, M. , Berman, D. , Perkova, M. , Grzybowski, T. , Lejrikh, A. , & Bulakhova, N. (2010). Phylogeography and molecular adaptation of Siberian salamander *Salamandrella keyserlingii* based on mitochondrial DNA variation. Molecular Phylogenetics and Evolution, 56, 562–571. https://doi.org/10.1016/j.ympev.2010.04.005 20398779 10.1016/j.ympev.2010.04.005

[ece34154-bib-0043] McClellan, D. A. , & Ellison, D. D. (2010). Assessing and improving the accuracy of detecting protein adaptation with the TreeSAAP analytical software. International Journal of Bioinformatics Research and Applications, 6, 120–133. https://doi.org/10.1504/IJBRA.2010.032116 20223735 10.1504/IJBRA.2010.032116

[ece34154-bib-0044] McClellan, D. A. , Palfreyman, E. J. , Smith, M. J. , Moss, J. L. , Christensen, R. G. , & Sailsbery, J. K. (2005). Physicochemical Evolution and Molecular Adaptation of the Cetacean and Artiodactyl Cytochrome b Proteins. Molecular Biology and Evolution, 22, 437–455. https://doi.org/10.1093/molbev/msi028 15509727 10.1093/molbev/msi028

[ece34154-bib-0045] McDevitt, A. D. , Mariani, S. , Hebblewhite, M. , DeCesare, N. J. , Morgantini, L. , Seip, D. , … Musiani, M. (2009). Survival in the Rockies of an endangered hybrid swarm from diverged caribou (*Rangifer tarandus*) lineages. Molecular Ecology, 18, 665–679. https://doi.org/10.1111/j.1365-294X.2008.04050.x 19175508 10.1111/j.1365-294X.2008.04050.x

[ece34154-bib-0046] Meier, J. I. , Sousa, V. C. , Marques, D. A. , Selz, O. M. , Wagner, C. E. , Excoffier, L. , & Seehausen, O. (2017). Demographic modelling with whole‐genome data reveals parallel origin of similar *Pundamilia* cichlid species after hybridization. Molecular Ecology, 26, 123–141. https://doi.org/10.1111/mec.13838 27613570 10.1111/mec.13838

[ece34154-bib-0048] Murrell, B. , Weaver, S. , Smith, M. D. , Wertheim, J. O. , Murrell, S. , Aylward, A. , … Scheffler, K. (2015). Gene‐wide identification of episodic selection. Molecular Biology and Evolution, 32, 1365–1371. https://doi.org/10.1093/molbev/msv035 25701167 10.1093/molbev/msv035 PMC4408417

[ece34154-bib-0049] Murrell, B. , Wertheim, J. O. , Moola, S. , Weighill, T. , Scheffler, K. , & Pond, S. L. K. (2012). Detecting individual sites subject to episodic diversifying selection. PLoS Genetics, 8, e1002764 https://doi.org/10.1371/journal.pgen.1002764 22807683 10.1371/journal.pgen.1002764 PMC3395634

[ece34154-bib-0050] Nagy, J. A. , Wright, W. H. , Slack, T. M. , & Veitch, A. M. (2005). Seasonal ranges of the Cape Bathurst, Bluenose‐West, and Bluenose‐East barren‐ground caribou herds. Canada: Department of Environmental & Natural Resources.

[ece34154-bib-0051] Nielsen, R. (2005). Molecular signatures of natural selection. Annual Review of Genetics, 39, 197–218. https://doi.org/10.1146/annurev.genet.39.073003.112420 10.1146/annurev.genet.39.073003.112420 16285858

[ece34154-bib-0052] Nosil, P. , Crespi, B. J. , & Sandoval, C. P. (2002). Host‐plant adaptation drives the parallel evolution of reproductive isolation. Nature, 417, 440–443. https://doi.org/10.1038/417440a 12024213 10.1038/417440a

[ece34154-bib-0053] Orr, H. A. (2005). The probability of parallel evolution. Evolution, 59, 216–220. https://doi.org/10.1111/j.0014-3820.2005.tb00907.x 15792240

[ece34154-bib-0054] Østbye, K. , Amundsen, P.‐A. , Bernatchez, L. , Klemetsen, A. , Knudsen, R. , Kristoffersen, R. , … Hindar, K. (2006). Parallel evolution of ecomorphological traits in the European whitefish *Coregonus lavaretus* (L.) species complex during postglacial times. Molecular Ecology, 15, 3983–4001. 17054498 10.1111/j.1365-294X.2006.03062.x

[ece34154-bib-0055] Palkovacs, E. P. , Dion, K. B. , Post, D. M. , & Caccone, A. (2008). Independent evolutionary origins of landlocked alewife populations and rapid parallel evolution of phenotypic traits. Molecular Ecology, 17, 582–597. 18179439 10.1111/j.1365-294X.2007.03593.x

[ece34154-bib-0056] Peischl, S. , Dupanloup, I. , Kirkpatrick, M. , & Excoffier, L. (2013). On the accumulation of deleterious mutations during range expansion. Molecular Ecology, 22, 5972–5982. https://doi.org/10.1111/mec.12524 24102784 10.1111/mec.12524

[ece34154-bib-0057] Perreault‐Payette, A. , Muir, A. M. , Goetz, F. , Perrier, C. , Normandeau, E. , Sirois, P. , & Bernatchez, L. (2017). Investigating the extent of parallelism in morphological and genomic divergence among lake trout ecotypes in Lake Superior. Molecular Ecology, 26, 1477–1497. https://doi.org/10.1111/mec.14018 28099784 10.1111/mec.14018

[ece34154-bib-0058] Pond, S. L. K. , & Frost, S. D. (2005). Datamonkey: Rapid detection of selective pressure on individual sites of codon alignments. Bioinformatics, 21, 2531–2533. https://doi.org/10.1093/bioinformatics/bti320 15713735 10.1093/bioinformatics/bti320

[ece34154-bib-0059] Pond, S. L. K. , Frost, S. D. , & Muse, S. V. (2005). HyPhy: Hypothesis testing using phylogenies. Bioinformatics, 21, 676–679. https://doi.org/10.1093/bioinformatics/bti079 15509596 10.1093/bioinformatics/bti079

[ece34154-bib-0060] Rambaut, A. (2016). FigTree v1.4.3. Retrieved from http://tree.bio.ed.ac.uk/software/figtree/

[ece34154-bib-0061] Rambaut, A. , Suchard, M. A. , Xie, D. , & Drummond, A. J. (2014). Tracer v1.6. Retrieved from http://beast.bio.ed.ac.uk/Tracer

[ece34154-bib-0062] Reid, S. D. , Herbelin, C. J. , Bumbaugh, A. C. , Selander, R. K. , & Whittam, T. S. (2000). Parallel evolution of virulence in pathogenic *Escherichia coli* . Nature, 406, 64–67. https://doi.org/10.1038/35017546 10894541 10.1038/35017546

[ece34154-bib-0063] Rettie, W. J. , & Messier, F. (2001). Range use and movement rates of woodland caribou is Saskatchewan. Canadian Journal of Zoology, 79, 1933–1940. https://doi.org/10.1139/z01-156

[ece34154-bib-0064] Røed, K. H. , Ferguson, M. A. D. , Crête, M. , & Bergerud, T. A. (1991). Genetic variation in transferrin as a predictor for differentiation and evolution of caribou from eastern Canada. Rangifer, 11, 65–74. https://doi.org/10.7557/2.11.2.979

[ece34154-bib-0065] Rokas, A. , & Carroll, S. B. (2008). Frequent and widespread parallel evolution of protein sequences. Molecular Biology and Evolution, 25, 1943–1953. https://doi.org/10.1093/molbev/msn143 18583353 10.1093/molbev/msn143

[ece34154-bib-0066] Seehausen, O. (2004). Hybridization and adaptive radiation. Trends in Ecology & Evolution, 19, 198–207. https://doi.org/10.1016/j.tree.2004.01.003 16701254 10.1016/j.tree.2004.01.003

[ece34154-bib-0067] Shafer, A. B. A. , Cullingham, C. I. , Côté, S. D. , & Coltman, D. W. (2010). Of glaciers and refugia: A decade of study sheds new light on the phylogeography of northwestern North America. Molecular Ecology, 19, 4589–4621. https://doi.org/10.1111/j.1365-294X.2010.04828.x 20849561 10.1111/j.1365-294X.2010.04828.x

[ece34154-bib-0068] Sim, Z. , Hall, J. C. , Jex, B. , Hegel, T. M. , & Coltman, D. W. (2016). Genome‐wide set of SNPs reveals evidence for two glacial refugia and admixture from postglacial recolonization in an alpine ungulate. Molecular Ecology, 25, 3696–3705. https://doi.org/10.1111/mec.13701 27272944 10.1111/mec.13701

[ece34154-bib-0069] Stewart, C.‐B. , Schilling, J. W. , & Wilson, A. C. (1987). Adaptive evolution in the stomach lysozymes of foregut fermenters. Nature, 330, 401–404. https://doi.org/10.1038/330401a0 3120013 10.1038/330401a0

[ece34154-bib-0070] Storz, J. F. (2016). Causes of molecular convergence and parallelism in protein evolution. Nature Reviews Genetics, 17, 239–250. https://doi.org/10.1038/nrg.2016.11 10.1038/nrg.2016.11 PMC5482790 26972590

[ece34154-bib-0071] Tajima, F. (1989). Statistical method for testing the neutral mutation hypothesis by DNA polymorphism. Genetics, 123, 585–595. 2513255 10.1093/genetics/123.3.585 PMC1203831

[ece34154-bib-0072] Tamura, K. , & Nei, M. (1993). Estimation of the number of nucleotide substitutions in the control region of mitochondrial DNA in humans and chimpanzees. Molecular Biology and Evolution, 10, 512–526. 8336541 10.1093/oxfordjournals.molbev.a040023

[ece34154-bib-0073] Tamura, K. , Stecher, G. , Peterson, D. , Filipski, A. , & Kumar, S. (2013). MEGA6: Molecular evolutionary genetics analysis version 6.0. Molecular Biology and Evolution, 30, 2725–2729. https://doi.org/10.1093/molbev/mst197 24132122 10.1093/molbev/mst197 PMC3840312

[ece34154-bib-0074] Taylor, E. B. , Foote, C. J. , & Wood, C. C. (1996). Molecular genetic evidence for parallel life‐history evolution within a Pacific salmon (sockeye salmon and kokanee, *Onorhynchus nerka*). Evolution, 50, 401–416. 28568856 10.1111/j.1558-5646.1996.tb04502.x

[ece34154-bib-0075] Thompson, C. E. , Taylor, E. B. , & McPhail, J. D. (1997). Parallel evolution of lake‐stream pairs of threespine sticklebacks (*Gasterosteus*) inferred from mitochondrial DNA variation. Evolution, 51, 1955–1965. 28565100 10.1111/j.1558-5646.1997.tb05117.x

[ece34154-bib-0076] Wada, K. , Nakamura, M. , Nishibori, M. , & Yokohama, M. (2010). The complete nucleotide sequence of mitochondrial genome in the reindeer (Rangifer tarandus) and red deer (Cervus elaphus). NCBI Reference Sequence, NCBI Genome Project.

[ece34154-bib-0077] Wang, G. , Zhai, W. , Yang, H. , Fan, R. , Cao, X. , Zhong, L. , … Poyarkov, A. D. (2013). The genomics of selection in dogs and the parallel evolution between dogs and humans. Nature Communications, 4, 1860 https://doi.org/10.1038/ncomms2814 10.1038/ncomms2814 23673645

[ece34154-bib-0078] Waples, R. S. , Teel, D. J. , Myers, J. M. , & Marshall, A. R. (2004). Life‐history divergence in Chinook salmon: Historic contingency and parallel evolution. Evolution, 58, 386–403. https://doi.org/10.1111/j.0014-3820.2004.tb01654.x 15068355

[ece34154-bib-0079] Weckworth, B. V. , Musiani, M. , McDevitt, A. D. , Hebblewhite, M. , & Mariani, S. (2012). Reconstruction of caribou evolutionary history in Western North America and its implications for conservation. Molecular Ecology, 21, 3610–3624. https://doi.org/10.1111/j.1365-294X.2012.05621.x 22612518 10.1111/j.1365-294X.2012.05621.x

[ece34154-bib-0080] Wicker, T. , Taudien, S. , Houben, A. , Keller, B. , Graner, A. , Platzer, M. , & Stein, N. (2009). A whole‐genome snapshot of 454 sequences exposes the composition of the barley genome and provides evidence for parallel evolution of genome size in wheat and barley. Plant Journal, 59, 712–722. https://doi.org/10.1111/j.1365-313X.2009.03911.x 19453446 10.1111/j.1365-313X.2009.03911.x

[ece34154-bib-0081] Wittmer, H. U. , McLellan, B. N. , Serrouya, R. , & Apps, C. D. (2007). Changes in landscape composition influence the decline of a threatened woodland caribou population. Journal of Animal Ecology, 76, 568–579. https://doi.org/10.1111/j.1365-2656.2007.01220.x 17439473 10.1111/j.1365-2656.2007.01220.x

[ece34154-bib-0082] Woolley, S. , Johnson, J. , Smith, M. J. , Crandall, K. A. , & McClellan, D. A. (2003). TreeSAAP: Selection on amino acid properties using phylogenetic trees. Bioinformatics Application Note, 19, 671–672. https://doi.org/10.1093/bioinformatics/btg043 10.1093/bioinformatics/btg043 12651734

[ece34154-bib-0083] Yannic, G. , Pellissier, L. , Ortego, J. , Lecomte, N. , Couturier, S. , Cuyler, C. , … Kolpashikov, L. (2014). Genetic diversity in caribou linked to past and future climate change. Nature Climate Change, 4, 132–137. https://doi.org/10.1038/nclimate2074

[ece34154-bib-0084] Yeager, M. , & Hughes, A. L. (1999). Evolution of the mammalian MHC: Natural selection, recombination, and convergent evolution. Immunological Reviews, 167, 45–58. https://doi.org/10.1111/j.1600-065X.1999.tb01381.x 10319250 10.1111/j.1600-065x.1999.tb01381.x

[ece34154-bib-0085] Yokoyama, R. , & Yokoyama, S. (1990). Convergent evolution of the red‐ and green‐like visual pigment genes in fish, *Astyanax fasciatus*, and human. Proceedings of the National Academy of Sciences, 87, 9315–9318. https://doi.org/10.1073/pnas.87.23.9315 10.1073/pnas.87.23.9315 PMC55155 2123554

